# Oxidative Stress and Antioxidant Pathway in Allergic Rhinitis

**DOI:** 10.3390/antiox10081266

**Published:** 2021-08-09

**Authors:** Munsoo Han, Dabin Lee, Sang Hag Lee, Tae Hoon Kim

**Affiliations:** Department of Otorhinolaryngology—Head and Neck Surgery, College of Medicine, Korea University, Seoul 02841, Korea; mshan35@gmail.com (M.H.); dabin425@korea.ac.kr (D.L.); sanghag@kumc.or.kr (S.H.L.)

**Keywords:** oxidative stress, antioxidants, allergic rhinitis

## Abstract

Oxidative stress is the cause and consequence of redox metabolism in various physiological and pathological conditions. Understanding the molecular pathways underlying oxidative stress and the role of antioxidants could serve as the key to helping treat associated diseases. Allergic rhinitis is a condition that deteriorates the daily function and quality of life of afflicted individuals and is associated with a high socioeconomic burden and prevalence. Recent studies have focused on the role of oxidative stress and antioxidants in allergic rhinitis. This review discusses animal and clinical studies on oxidative markers and the potential therapeutic dietary antioxidants for allergic rhinitis.

## 1. Introduction

Oxidative stress is the cause of a variety of physiological and pathological conditions. An increase in the levels of oxidants may be overwhelming to the natural antioxidant system, leading to direct cellular damage or aberrations in molecular signaling pathways [1]. As scientific research has focused on identifying the molecular pathways underlying oxidative stress, several potential therapeutic antioxidants have been studied. These antioxidants are drawing attention because conventional medical therapies have been inefficient in completely curing a number of diseases.

In that sense, oxidative stress has been studied in conditions such as asthma, one of the respiratory tract inflammatory diseases, severe cases of which are difficult to treat by conventional medical therapies. The role of various biomarkers of oxidative stress, such as nitrotyrosine (Tyr-NO2) and nuclear factor erythroid 2-related factor 2 (Nrf2) in asthma, has been investigated [2,3]. Meanwhile, it is believed that upper and lower airway diseases such as allergic rhinitis, chronic rhinosinusitis, and asthma often co-exist (the “one airway concept”) [4]. The majority of asthma patients have allergic rhinitis, and many patients with rhinitis have asthma [5,6,7]. Considering that allergic rhinitis and asthma may share similar pathophysiology, a theory investigated in asthma is often studied in allergic rhinitis.

Allergic rhinitis is a common health problem characterized by watery rhinorrhea, nasal obstruction, nasal pruritus, and sneezing [8]. It is reported to occur in a great number of people worldwide, which is more than 500 million people across the globe, including approximately 30% of the population in Western countries, and its high prevalence continues to grow [5,8,9,10]. Allergic rhinitis causes a considerable economic burden, with an estimated annual cost of approximately 2–5 billion USD in the United States alone [11]. It is a risk factor for asthma exacerbation [12]. Allergic rhinitis is a critical illness that exerts a negative impact not only on socioeconomic costs but also on an individual’s functioning in school or work, sleep, and quality of life [13,14].

Considering the negative influence on health, high prevalence, and socioeconomic cost of allergic rhinitis, its efficient management and control of the disease are crucial. According to the Allergic Rhinitis and its Impact on Asthma (ARIA) guidelines, management of allergic rhinitis includes patient education, medical therapy, and allergen-specific immunotherapy [8]. However, pharmacotherapy for allergic rhinitis, including oral antihistamines and intranasal corticosteroid sprays, is limited in effect, and up to 29% of children and 62% of adults report partial or poor relief with medical therapy alone [15,16]. While treatments such as immunotherapy are reported to be effective in reducing symptoms and the requirement for rescue medication in patients with allergic rhinitis, data on their long-term effectiveness are lacking [17].

Given the current situation in the management of allergic rhinitis, several complementary and alternative medicines have been investigated for the treatment of allergic rhinitis [8]. Antioxidant therapy has recently been applied for allergic rhinitis treatment. As the role of oxidative stress and antioxidants in the pathophysiology of asthma has drawn attention for research, oxidative stress in allergic rhinitis has also been studied because allergic rhinitis is linked to asthma. These studies are expected to improve our understanding of allergic rhinitis and encourage the development of novel therapeutic options. In this article, we have reviewed the current knowledge on the molecular pathways of oxidative stress and antioxidants in allergic rhinitis and the potential therapeutic options with dietary antioxidants for allergic rhinitis.

## 2. Oxidative Stress Pathways

### 2.1. Overview of Oxidative Stress

**Figure 1:**
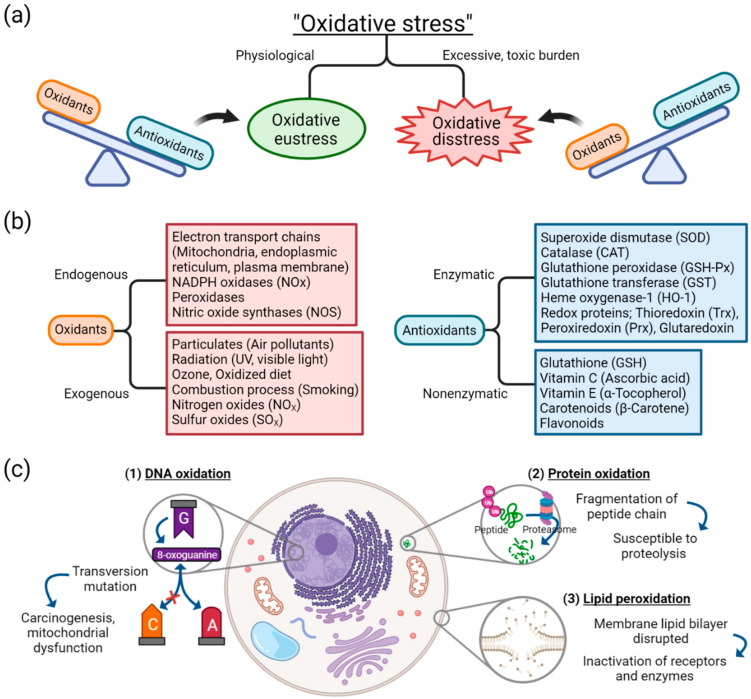
Basic concepts and components of oxidative stress: (**a**) oxidative stress is distinguished as either oxidative eustress or oxidative distress, depending on the balance between oxidants and antioxidants; (**b**) sources of oxidants and antioxidants; (**c**) effect of oxidative damage on biomolecules of DNA, proteins, and lipids. Abbreviation: DNA, deoxyribonucleic acid.

Oxidative stress arises from oxidation-reduction (redox) homeostasis. The global concept of oxidative stress is defined as “an imbalance between oxidants and antioxidants in favor of the oxidants, leading to a disruption of redox signaling and control and/or molecular damage” [1,18]. The concept of ranging oxidative stress in terms of intensity was introduced [19,20]. When the balance between oxidants and antioxidants is maintained in a steady-state redox balance, in which stress from oxidants does not overweigh antioxidants, it is of a reversible and physiological state and thus called “oxidative eustress” (Figure 1a) [1]. Oxidative eustress is an important concept in redox control, and it acts as physiological redox signaling [21]. In contrast, when the balance between oxidants and antioxidants deviates to the direction of oxidants, due to excessive and supraphysiological exposure of oxidants or malfunction of antioxidant defense mechanism, aberrant redox signaling or molecular damage occurs, which is termed “oxidative distress”.

In oxidative stress, free radicals such as reactive oxygenated species (ROS) are generated by different sources. Despite the complexity in understanding the molecular pathways and biochemical components involved in redox signaling and stress response to oxidants, different sources of oxidants have been identified. As depicted in Figure 1b, diverse endogenous sources of oxidants operate in cells and produce reactive species as normal cellular metabolism, which, in adequate quantity, are essential in cell homeostasis, gene expression, and signal transduction [22]. Opposite to oxidants are antioxidants, which can be categorized into either enzymatic or nonenzymatic [23]. The antioxidant system is associated with counteracting the effects of oxidants. When the endogenous or exogenous source of oxidants is produced in greater quantity or the antioxidant defense mechanism is decreased, oxidative distress occurs. For example, among exogenous sources of oxidants, cigarette smoke is known to contain free radicals, including superoxide and nitric oxide, thus capable of inducing oxidative stress injury in airway epithelium [24].

Cellular injury as an effect of oxidative stress can be classified into three categories: damage to nucleic acids, proteins, or lipids (Figure 1c). Oxidation of deoxyribonucleic acids (DNA) can contribute to instability of the genome. One of the examples of DNA oxidation, guanine, among the DNA bases, is most susceptible to oxidative stress [25]. It is transformed into a mutagenic lesion, 8-oxoguanine, after oxidative damage. Instead of paring with cytosine, 8-oxoguanine base pairs with adenine, thus producing transversion mutation when replicated. This oxidatively modified biomolecule is capable of causing mitochondrial dysfunction and tumorigenesis [26].

Oxidation of protein generates oxidation products of amino acid side chains [27]. ROS is capable of causing peptide chain fragmentation, change of electrical charge of proteins, and oxidation of specific amino acids [23,28]. This means proteins are susceptible to degradation by proteases, and the oxidatively modified enzymes show decreased activities [29]. Especially among the amino acids, cysteine and methionine are more susceptible to oxidation, and enzymes with metal elements are more sensitive to metal-catalyzed oxidation.

One of the oxygen-derived free radicals is peroxyl radicals (ROO–•); it acts in the peroxidation of fatty acids [23]. Free radicals trigger chain reactions of lipid peroxidation, generated lipid radical reacts with oxygen, and peroxyl radicals are produced. Then, peroxyl radical transforms polyunsaturated fatty acids into lipid hydroperoxides, which are unstable and disintegrated into unsaturated aldehydes or malondialdehydes (MDAs). MDAs are one of the commonly used oxidative stress markers [2,30], and are capable of forming cross-linkages of proteins and thus inactivating them [31]. The lipid peroxidation counteracts cell membrane integrity itself, disrupting the membrane lipid bilayer and downregulating membrane receptors and enzymes.

### 2.2. Oxidative Stress and Possible Therapeutic Antioxidants in Diseases

**Figure 2:**
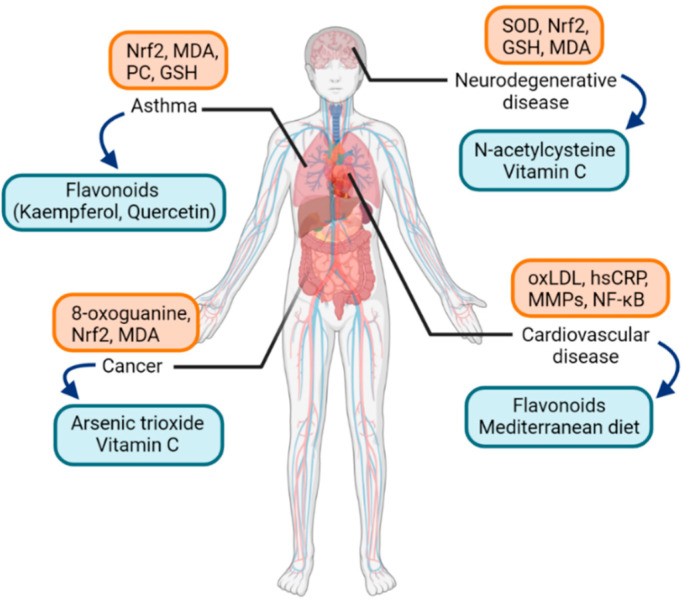
Examples of oxidative-stress-related diseases and associated oxidative stress markers (orange box) along with possible therapeutic antioxidants (blue box). Abbreviations: Nrf2, nuclear factor erythroid 2-related factor 2; MDA, malondialdehyde; PC, protein carbonyl; GSH, glutathione; SOD, superoxide dismutase; oxLDL, oxidized low-density-lipoprotein; MMPs, matrix metalloproteinases; NF-κB, nuclear factor kappa-light-chain-enhancer of activated B cells.

A great amount of health and disease states have been covered in the literature regarding the field of molecular pathways of oxidative stress. Biomarkers of oxidative stress, either protein, lipid, or DNA, have been analyzed in numerous diseases in humans. Some studies examined if specific oxidative markers are related to specific diseases, and cluster analysis was performed to compare the clinical relevance of oxidative stress markers and the correspondence among diseases [2]. Among them, studies regarding asthma are worth attention. As for biomarkers of oxidative stress in asthma, in a recent study, oxidative stress and antioxidant capacity were measured in asthma patients and healthy individuals [32]. MDA and protein carbonyl (PC) levels were significantly increased in asthma patients, but glutathione (GSH) levels were decreased (Figure 2).

Asthma is a relatively common disease characterized by airway hyperresponsiveness and airway inflammation and remodeling. The pathogenesis of asthma involves activation of various inflammatory cell infiltration and production of different cytokines [33]. In the pathogenesis of asthma, intracellular signaling cascades involving Toll-like receptors (TLRs) and transcription factors such as nuclear factor kappa-light-chain-enhancer of activated B cells (NF-κB) are important [34]. Potent exogenous oxidants such as cigarette smoke and ozone increase the expression of TLRs and contribute to the redox balance in the lungs [35,36]. Furthermore, as a redox-sensitive transcription factor, NF-κB is activated by ROS to result in chromatin remodeling and expressing proinflammatory mediators [37,38]. Another transcription factor, Nrf2, regulates antioxidant response, including maintenance of epithelial barrier integrity and proliferation in smooth muscle cells in the airway, and was found to exert aberrant activity in asthma [39,40].

While conventional therapy of asthma comprises inhaled or systemic corticosteroids, inhaled β2-adrenoceptor agonists, and leukotriene receptor antagonists [41], the effectiveness of these conventional treatment methods is rather unsatisfactory in patients with severe asthma [42,43]. The need for a novel alternative therapeutic option could be met by targeting the signaling pathways of oxidative stress in asthma [34]. Among the studies on antioxidants in asthma, yielding relatively strong antioxidant properties are dietary flavonoids. Flavonoids have antioxidant activities to regulate cellular signaling pathways involving transcription factors such as NF-κB. Examples of flavonoids are quercetin and kaempferol, and especially the latter showed to alleviate airway inflammation in an animal allergic asthma model [44]. A limited number of clinical trials have been promoted for flavonoids in asthma patients and showed improvements in serum leukotriene levels, peak expiratory flows, or asthma symptom scores [45,46]. As allergic rhinitis shares similar pathogenesis as asthma in terms of airway inflammation, encouraging results of studies about antioxidants in asthma gives a direction of research for allergic rhinitis.

As for other diseases known to be associated with oxidative stress, Alzheimer’s disease is one of the most prevalent neurodegenerative diseases. It shows symptoms of dementia, impaired spatial memory, and cognitive deficits [47]. It has been reported that DNA damage, lipid peroxidation, and protein nitration are increased in Alzheimer’s disease [48]. Another significant neurodegenerative disease is autism. Autism spectrum disorders are neurodevelopmental disorders with impairments in social interaction, language, perception, and behaviors. Mitochondrial dysfunction represented by decreased expression of electron transport complexes and superoxide dismutase (SOD) in mitochondria was noted, leading to elevated production of ROS [49]. Possible antioxidant treatment to reduce oxidative stress and improve mitochondrial function includes ascorbic acid and N-acetylcysteine [50,51].

In cardiovascular diseases, elevated production of ROS leads to oxidative damage and is known to worsen ischemia-reperfusion injury in myocardial infarction [52]. Atherosclerosis, as the leading cause of mortality from cardiovascular disease, is also affected by inflammatory mechanisms derived from oxidative stress [53]. Oxidized cholesterol, or oxysterol, including oxidatively modified low-density lipoprotein (oxLDL) molecules, activates endothelial cells by releasing bioactive phospholipids, and then circulating monocytes differentiate into macrophages, which is an important pathogenesis in atherosclerosis [54,55]. Additionally, protein biomarkers, such as high sensitivity C-reactive protein (hsCRP) or matrix metalloproteinases (MMPs), and transcriptional factors such as NF-κB, are well-known in the pathogenesis of atherosclerosis; hsCRP binds to oxLDL and promotes endothelial dysfunction [56]. As for therapeutic antioxidants, there is still controversy about whether antioxidants are an effective treatment modality in cardiovascular diseases. Recent reviews and meta-analyses showed results of reduced mortality due to cardiovascular diseases by supplementing antioxidants such as flavonoids, green teas, or the Mediterranean diet [57,58,59]. Experimental studies identified that oxidant and proinflammatory mediators such as ROS and H_2_O_2_ are produced in cardiac tissues and murine macrophages, and the antioxidant resveratrol reduced the production of the oxidants [60].

In terms of malignancy, various redox-oriented cancer therapies have been evaluated in clinical settings [61,62]. One of the agents with potential redox activity that showed a promising result with US Food and Drug Administration (FDA) approval for acute promyelocytic leukemia is arsenic trioxide [63]. Vitamin C was studied in colon cancer; at high doses, it acts as an oxidant, yielding potential anticancer activity [64]. Other anticancer drugs with potential clinical activities were covered in a recent review [65].

## 3. Allergic Rhinitis and Oxidative Stress

### 3.1. Pathophysiology of Allergic Rhinitis

**Figure 3:**
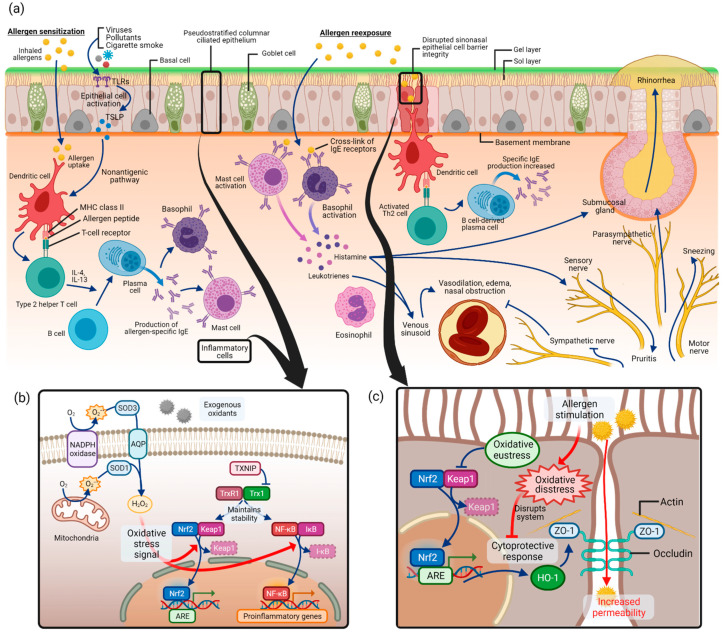
Allergic rhinitis and oxidative stress pathways: (**a**) pathogenesis of allergic rhinitis as in allergic sensitization followed by allergen reexposure; (**b**) regulation of signaling pathways by Nrf2 and NF-κB in oxidative stress of allergic rhinitis; (**c**) disrupted sinonasal epithelial cell barrier integrity in allergic rhinitis and associated oxidative stress pathway. Abbreviations: NADPH, nicotine adenine dinucleotide phosphate; SOD, superoxide dismutase; AQP, aquaporin; H_2_O_2_, hydrogen peroxide; TXNIP, thioredoxin-interacting protein; TrxR1, thioredoxin reductase 1; Trx1, thioredoxin 1; Nrf2, nuclear factor erythroid 2-related factor 2; Keap1, Kelch-like ECH-associated protein 1; NF-κB, nuclear factor kappa-light-chain-enhancer of activated B cells; IκB, inhibitor of NF-κB; ARE, antioxidant response element; HO-1, heme oxygenase-1; ZO-1, zonula occludens-1.

Allergic rhinitis is an immunoglobulin E (IgE)-mediated inflammation of the nasal mucosa induced by allergen inhalation [8,66]. A variety of components involving cells of the nasal cavity and inflammatory cells, cytokines, mediators, and cell adhesion molecules participate in the process of allergic rhinitis. As depicted in Figure 3a, the pathogenesis of allergic rhinitis begins with allergen sensitization [12,67]. Inhaled allergens in the nasal epithelium are captured by dendritic cells, which act as antigen-presenting cells, and are presented as allergenic peptides to T lymphocytes [68]. This induces T helper 2 (Th2) cells, which secrete Th2 type cytokines such as interleukin (IL)-4, IL-5, IL-10, and IL-13. This process converts B lymphocytes into allergen-specific IgE-producing plasma cells. The released allergen-specific IgE molecules bind to tissue mast cells and circulating basophils.

Upon allergen reexposure, the same allergen binds to the surface IgE on mast cells and basophils and activates the cells. Various kinds of neuroactive and vasoactive mediators, such as histamine and leukotriene, are released from the cells. Typical allergic rhinitis symptoms, such as nasal congestion, rhinorrhea, or itching, are produced by substances that act on vessels and glands of the nose. In addition, Th2 lymphocytes are activated by dendritic cells, and consequently release cytokines and chemokines, which mediate the recruitment of inflammatory cells such as T cells, B cells, eosinophils, basophils, and neutrophils to the nasal mucosa, further progressing allergic rhinitis reactions.

### 3.2. Oxidative Stress and Allergic Rhinitis

Most disease states include redox component at some degree or stage, and it is important to understand the complex multifactorial concept of oxidative stress that in a disease state, oxidant and antioxidant factors could act at the same time, leading to oxidative eustress or distress depending on which factor is stronger [1]. It also helps to note that the antioxidant system can operate in a paradoxical way from what was expected, according to its quantity, stage, or tissue [69,70].

Due to its complexity and multifactorial components in understanding the molecular pathways and signals of oxidative stress, and since allergic rhinitis is a relatively less explored topic for studying oxidative stress, recent studies regarding oxidative stress in allergic rhinitis have focused on identifying the change of the quantity of known oxidants and antioxidants in allergic rhinitis states and after treatment with dietary antioxidants. After reviewing the literature on oxidative stress pathways and the potential therapeutic antioxidants in allergic rhinitis, we combined the results of the studies with previously known molecular pathways of oxidative stress, as schematically shown in Figure 3.

ROS production by mitochondria and nicotine adenine dinucleotide phosphate (NADPH) oxidase was first discovered in phagocytes but later turned out to be present in different epithelial and inflammatory cells (Figure 3b) [1]. Superoxide anion (O_2_–•) is produced by adding one electron to molecular oxygen O_2_, mediated by the mitochondrial electron transport system or NADPH oxidase [23,71]. Approximately 1–3% of electrons leak from the mitochondrial electron transport system and produce superoxide instead of being reduced [23]. Then, superoxide is converted to hydrogen peroxide (H_2_O_2_) by SODs. Extracellular H_2_O_2_ is captured and imported into the cytoplasm by aquaporins (AQPs) [72]. As a major signaling molecule in redox signaling, H_2_O_2_ acts as a messenger in a variety of oxidative eustress (physiologic, health condition) and distress (pathologic condition) pathways [21]. It is capable of regulating the activity of several transcription factors such as Nrf2 and NF-κB [73].

As commonly known signaling pathways in oxidative stress, Nrf2/Keap1 (Kelch-like ECH-associated protein 1) and NF-κB pathways have been covered in allergic rhinitis. What is known in general is that Nrf2/Keap1 and NF-κB pathways serve as “molecular redox switches” which control activation or deactivation cycles and modulate system activities in a broad range of biological conditions [1] (p. 730). In unstressed conditions, Nrf2 is suppressed in transcriptional function due to ubiquitination and degradation by Keap1 [74]. Under oxidative stress, Keap1 is modified and no longer capable of ubiquitinating Nrf2, leading to the release of Nrf2 and its accumulation in the nucleus, which acts as a transcription factor to induce antioxidant and detoxication enzymes [75]. In this signaling cascade, thioredoxin reductase 1 (TrxR1) was reported to be a regulator for Nrf2 [76].

For allergic rhinitis, the role of thioredoxin-interacting protein (TXNIP) was evaluated in oxidative stress [77]. In the ovalbumin (OVA)-induced allergic rhinitis murine model, expression of TXNIP in nasal mucosa, MDA level, and SOD activity were measured, along with allergic rhinitis markers such as nasal symptoms of sneezing and nasal rubbing, OVA-specific IgE and histamine in serum, and OVA-specific IgE, IL-4, IL-5, and tumor necrosis factor (TNF)-α in the nasal lavage fluid. The outcomes were measured between mice that received intranasal administration of a TXNIP inhibitor, resveratrol, and those without treatment. In the untreated allergic rhinitis group, nasal symptoms, TXNIP and OVA-specific IgE levels, histamine and cytokine levels, and MDA levels were increased, and SOD level was decreased, but these results were attenuated in the resveratrol-treated group. In the nasal tissue, epithelial cells and inflammatory cells were found to be TXNIP-positive. This result implies that the regulation of transcription factors of oxidative stress pathway by thioredoxin and TXNIP is relevant in allergic rhinitis, although the understanding of the exact signaling pathway between the transcription factors and allergic rhinitis markers such as cytokines, inflammatory cells, and nasal symptoms warrants further research.

Another transcription factor, NF-κB, is also capable of being activated in response to oxidative stress. After oxidation by H_2_O_2_, the inhibitory subunit of the NF-κB inhibitor (IκB) is released [78]. Then, NF-κB is freed and allowed to enter the nucleus, acting as a transcription factor and expression of genes involved in inflammatory, immune, or acute-phase responses [79,80,81].

In allergic rhinitis, several recent studies covered the activity of the NF-κB pathway in the allergic rhinitis murine model [82,83]. It was reported in the studies that in the OVA-induced allergic rhinitis model, oxidative stress markers such as MDA level and Nrf2 and NF-κB pathways are upregulated. They were associated with inflammatory signs such as cytokine levels and histopathology findings in allergic rhinitis models. After treatment with an antioxidant, mangiferin, the markers were downregulated.

In the pathogenesis of allergic rhinitis, antigen presentation by dendritic cells is the first step in allergen sensitization. It has been reported that physical epithelial barrier dysfunction in the nasal epithelium may contribute to the uptake of allergens and harmful exogenous particles in allergic rhinitis (Figure 3c) [84,85,86]. In allergic rhinitis patients with house dust mite allergy, epithelial barrier function impairment was found with increased epithelial permeability and altered occludin and zonula occludens (ZO)-1 expression [87]. This disrupted mucosal integrity could contribute to the decreased response to medical therapy, making it an important study area for the treatment of allergic rhinitis. Several studies have investigated the role of oxidative stress in epithelial cell barrier dysfunction in allergic rhinitis (Figure 3c).

The nasal epithelial cell barrier is made up of cell-to-cell tight junctions, which are formed with scaffold adaptor proteins ZO-1, ZO-2, and ZO-3 and integral membrane proteins such as occludin [86,88]. In a recent study, human sinonasal epithelial cells were used to study epithelial cell barrier function under allergic conditions [89]. After stimulation with house dust mite, the authors stained epithelial cells for the epithelial cell junction protein ZO-1 and measured epithelial cell permeability. Stimulation with house dust mite resulted in global disruption of ZO-1 and increased permeability in sinonasal epithelial cells.

Another study used the OVA-induced allergic rhinitis mouse model and investigated various outcomes, including epithelial cell permeability, after administration of a possible antioxidant, *Piper nigrum* extract [90]. By enhancing the Nrf2 transcription factor pathway, anti-inflammation enzyme heme oxygenase (HO)-1 synthesis was increased. As a consequence, ZO-1 and occludin degradation was inhibited, and the epithelial barrier integrity was enhanced.

According to the previous experimental and clinical studies which we have reviewed above, several oxidative stress markers were identified and found to be increased in allergic rhinitis. It is difficult to say that the markers are specific to allergic rhinitis since NF-κB and Nrf2 pathways are also found in oxidative stress pathways in other inflammatory disease conditions. Nrf2 is a well-known, clinically relevant biomarker of oxidative stress in diseases such as chronic obstructive pulmonary disorder, cancer, or Alzheimer’s disease [91,92,93]. Because a number of oxidative stress markers are relevant in various diseases and similar diseases might share the same relevant oxidative stress markers, future researchers can refer to biomarkers of asthma, which share similar pathogenesis to allergic rhinitis [2].

### 3.3. Therapeutic Antioxidants in Allergic Rhinitis

Most of the studies regarding oxidative pathways in allergic rhinitis have focused on finding potential dietary antioxidants as an alternative pharmacotherapy option for controlling the disease. Therefore, current literature is somewhat limited in defining the complex and diverse molecular pathways of oxidative stress specific to allergic rhinitis. Recent studies which dealt with those dietary antioxidants in allergic rhinitis were reviewed, and the details, including the natural diet source of each antioxidant, are described in Table 1 [77,82,83,89,94,95,96,97,98].

In several studies, the effect of sulforaphane was evaluated. A study in 2017 evaluated nasal epithelial cell barrier function after administration of sulforaphane [89]. In allergic rhinitis condition with house dust mite, epithelial junction protein, zonula occludens (ZO)-1 was decreased but restored after Nrf2 activation by sulforaphane. This was the first study to show that nasal epithelial cell barrier dysfunction in allergic rhinitis can be inhibited by activation of the Nrf2 pathway by sulforaphane treatment. Additionally, in a double-blinded, randomized, placebo-controlled clinical trial, sulforaphane showed effects in decreasing T2 cytokines such as IL-4, IL-5, and IL-13 in nasal cavity mucus of allergic rhinitis patients [94]. Subjective and objective parameters of allergic rhinitis such as total nasal symptom score (TNSS) and peak nasal inspiratory flow (PNIF) were improved after 3 weeks of sulforaphane treatment.

As mentioned in the previous section, the allergic rhinitis animal model study showed the effectiveness of resveratrol in reducing oxidative stress markers such as MDA [77]. TXNIP level was positively correlated with MDA levels but negatively correlated with SOD activities, implying that resveratrol treatment might decrease TXNIP levels, leading to attenuation of oxidative stress. Resveratrol was tested in a double-blinded, randomized, placebo-controlled study; patients who were administered with resveratrol showed a decrease in nasal symptoms, serum IgE, IL-4, TNF- α, and eosinophil levels, compared to the placebo group [95]. The clinical effect of resveratrol in decreasing nasal symptoms has been reported before [99].

A mango extract, mangiferin, has also been the target in some studies. In the OVA-induced murine allergic rhinitis model, mice that were administered with mangiferin showed fewer nasal symptoms and nasal mucosa inflammation, and inflammatory cell infiltration and epithelial disruption were reduced in histopathology. In nasal lavage fluid (NALF), after mangiferin treatment, MDA level was reduced, SOD activity was increased, and Nrf2/HO-1 expression was upregulated, while expression of NF-κB was decreased [82]. In another recent study with an allergic rhinitis animal model, after administration of mangiferin, MDA generation after allergen exposure was decreased. NF-κB signaling pathway activation was prevented, leading to the downregulation of inflammatory cytokines such as TNF-α and IL-1β. In histology, allergic rhinitis-related nasal epithelial changes such as ciliary loss and eosinophil infiltration were attenuated [83].

As described above, the study with a potential therapeutic antioxidant *Piper nigrum* extract evaluated the effect in the OVA-induced allergic rhinitis murine model [90]. The mice were orally administered with either *Piper nigrum* extract or dexamethasone. In mice treated with *Piper nigrum* extract, histamine release from mast cells, nasal symptoms, and eosinophil infiltration in nasal lavage fluid and nasal tissue were decreased. The result suggested that the antioxidant treatment promoted the cytoprotective function of the Nrf2 and heme oxygenase (HO)-1 signaling pathway, which resulted in inhibiting the disruption of tight junction proteins in the allergic rhinitis model.

Quercetin, a flavonoid, is known to yield a strong antioxidant property compared to other natural antioxidants. In allergic rhinitis patients, sinonasal epithelial cells were harvested and used to find their effectiveness [97]. In the study, after quercetin treatment, thioredoxin (TRX) production was increased in ELISA in response to H_2_O_2_ stimulation. Additionally, in OVA-sensitized mice, quercetin administration led to inhibition of nasal symptoms in allergic rhinitis mice. In nasal lavage fluids obtained 6 h after allergen challenge, TRX levels were increased.

Lastly, taurine was studied in allergic rhinitis patients and in the OVA-induced murine allergic rhinitis model [98]. Taurine administration showed a decrease in SOD3 level, nasal symptoms, inflammatory cytokine production, and inflammatory cell infiltration.

## 4. Conclusions

To study the effect of oxidative stress, transcriptional factors such as Nrf2 and NF-κB have been investigated in murine models of allergic rhinitis as well as nasal mucosa epithelial cells of patients with allergic rhinitis. Several possible therapeutic antioxidants that are abundant in natural dietary sources have been studied and have shown promising results by inhibiting several oxidative stress pathway markers. As limitations exist with currently used treatment methods in allergic rhinitis patients, a more detailed understanding of oxidative stress and antioxidants in allergic rhinitis would lead to better control of the disease. Future studies with therapeutic antioxidants could focus on clinical studies of allergic rhinitis patients based on previous literature.

## Figures and Tables

**Table 1:** Recent studies regarding the potential therapeutic antioxidants in relation to oxidative stress in allergic rhinitis.

Exogenous Antioxidants	Diet Source	Authors (Year)	Study Designs	Therapeutic Antioxidant Effects
Sulforaphane	Broccoli, cabbage	Yusin, J. et al. (2021) [94]	Clinical trial (double-blind, randomized, placebo-controlled)	Clinical measurements (TNSS, PNIF) of AR patients improved after sulforaphane supplementation. In nasal mucus fluid, T2 cytokines such as IL-4, IL-5, and IL-13 were decreased, but there was no statistical significance.
		London, N.R., Jr. et al. (2017) [89]	Human study (tissue-specific)	Human SNECs were harvested and stimulated with HDM with/without Nrf2 activation with sulforaphane. Epithelial cell junction protein ZO-1 was disrupted with HDM stimulation but increased when treated with sulforaphane before stimulation with HDM. Similar beneficial effect was found with transepithelial electrical resistance.
Resveratrol	Grapes, berries, peanuts	Zhang, W. et al. (2020) [77]	Animal study (OVA-induced murine AR model)	After resveratrol treatment, TXNIP, MDA, SOD, inflammatory cytokines, eosinophil numbers, and nasal symptoms were significantly altered compared to untreated AR mice.
		Lv, C. et al. (2018) [95]	Clinical trial (double-blind, randomized, placebo-controlled)	AR patients treated with resveratrol showed reduction in nasal symptoms, serum IgE, IL-4, TNF-α, and eosinophil levels.
Mangiferin	Mango	Piao, C.H. et al. (2020) [82]	Animal study (OVA-induced murine AR model)	Mangiferin treatment led to reduction of nasal symptoms, nasal mucosa inflammation, inflammatory cell infiltration, and epithelial disruption in histopathology. In NALF, MDA level reduced, SOD activity increased, and Nrf2/HO-1 expression was upregulated, while expression of NF-κB was decreased.
		Wang, Y. et al. (2020) [83]	Animal study (OVA-induced murine AR model)	After administration of mangiferin, MDA level was decreased, and NF-κB pathway was prevented, which led to downregulation of TNF-α and IL-1β. In histopathology, ciliary loss and eosinophil infiltration were decreased.
*Piper nigrum* extract	Black pepper	Bui, T.T. et al. (2020) [90]	Animal study (OVA-induced murine AR model)	After *Piper nigrum* extract treatment, mast cells histamine release, nasal symptoms in early phase reaction, and eosinophil accumulation in nasal lavage fluid and nasal tissue were decreased.
Quercetin	Onions, red wine, tea	Edo, Y. et al. (2018) [97]	Human study (tissue-specific) Animal study (OVA-induced murine AR model)	Human SNECs showed increased TRX production in ELISA when treated with quercetin. In animal model, quercetin was orally administered, and the nasal symptoms were inhibited. In NALF, TRX levels were increased.
Taurine	Scallops, tuna, octopus	Zhou, J. et al. (2020) [98]	Human study (serum marker) Animal study (OVA-induced murine AR model)	In AR patients compared to healthy controls, after treatment of taurine, serum SOD3 level was decreased. In animal model, AR symptoms, inflammatory cytokines (TNF- α, IL-4, and IL-6), and eosinophil and mast cell infiltration in nasal mucosa were decreased. SOD3 production was increased.

Abbreviations: TNSS, total nasal symptom score; PNIF, peak nasal inspiratory flow; AR, allergic rhinitis; IL, interleukin; SNEC, sinonasal epithelial cell; HDM, house dust mite; Nrf2, nuclear factor erythroid 2-related factor 2; ZO-1, zonula occludens-1; OVA, ovalbumin; TXNIP, thioredoxin-interacting protein; MDA, malondialdehyde; SOD, superoxide dismutase; IgE, immunoglobulin E; TNF-α, tumor necrosis factor-α; NALF, nasal lavage fluid; HO-1, heme oxygenase-1; NF-κB, nuclear factor kappa-light-chain-enhancer of activated B cells; ELISA, enzyme-linked immunosorbent assay; TRX, thioredoxin.
